# Clozapine Re-challenge in a Patient With Pericarditis: A Case Report

**DOI:** 10.7759/cureus.87562

**Published:** 2025-07-08

**Authors:** Aliu O Yakubu, Laura Inglis, Sudhir Vusikala

**Affiliations:** 1 General Adult Psychiatry, University Hospital Wishaw, Wishaw, GBR

**Keywords:** antipsychotics, cardiotoxicity, clozapine, clozapine re-challenge, pericarditis

## Abstract

Pericarditis is a rare but potentially severe adverse event associated with clozapine treatment, which often results in clozapine discontinuation. There is limited evidence or guidance relating to the safety of reintroducing clozapine in patients who have developed pericarditis. In this report, we describe a successful clozapine re-challenge in a 53-year-old male patient with treatment-resistant schizophrenia who developed pericarditis after many years of stability on clozapine. He was admitted with a sudden onset of pleuritic and positional chest pain and was found to have widespread saddling ST elevation and elevated troponin and inflammatory markers. The echocardiogram demonstrated pericarditis and a small pericardial effusion. Treatment was initiated for pericarditis, and clozapine was suspended. Following symptom resolution, the patient was transferred to inpatient psychiatry, where clozapine reintroduction began on day 9 of admission. A standard clozapine titration schedule was followed over 15 days with weekly monitoring. Clozapine was successfully re-titrated without any clinical, biochemical, or radiological deterioration of pericarditis. This case highlights the safe and successful re-titration of clozapine in patients presenting with pericarditis, within weeks of initial presentation and with ongoing mild biochemical disturbances without any significant deterioration in cardiovascular status or function.

## Introduction

Clozapine is an atypical antipsychotic often used for treatment-resistant schizophrenia and other severe psychiatric conditions where first- and second-line antipsychotic treatments have failed [1]. Despite its efficacy, clozapine is associated with a range of potentially severe adverse effects, including cardiovascular complications such as pericarditis, pulmonary embolism, QT prolongation, and sudden cardiac death, as well as metabolic syndrome [2]. The exact mechanism behind clozapine-induced cardiotoxicity remains unclear, though several hypotheses have been proposed. One explanation suggests that clozapine may cause a delayed hypersensitivity reaction, acting as a hapten to trigger an immune response that can progress to pericarditis [3]. The management of clozapine treatment after an episode of cardiovascular complications presents a clinical challenge, particularly in balancing the risk of recurrence against the therapeutic benefits of the medication [4]. This case report discusses successful clozapine re-challenge in a patient who developed pericarditis during treatment, highlighting the clinical course, decision-making process, and outcomes. By documenting this case, we aim to contribute to the limited literature on the management of clozapine challenge following pericarditis, providing insight for future clinical practice.

## Case presentation

A 53-year-old male patient was admitted under acute medicine with chest pain. He had a background of paranoid schizophrenia, which had remained stable on clozapine 200 mg mane and 500 mg nocte for more than 20 years. On admission, he described a sudden onset of left-sided chest pain at rest, with no radiation or autonomic features, which was worse on inspiration and improved on sitting forward. Initial investigations included a negative D-dimer, normal thyroid function tests, normal urea and electrolytes, normal liver function tests (LFTs), and an unremarkable chest X-ray. The white cell count was elevated at 19.8 x 10^9^/L with predominant neutrophils at 17.3 x 10^9^/L. Mild elevations in C-reactive protein (CRP) and troponin T were noted at 16 mg/L and 14 ng/L, respectively. The ECG demonstrated widespread concave ST elevation. Cardiology was consulted and advised a likely diagnosis of pericarditis. Treatment was initiated with colchicine 500 mcg twice daily and regular ibuprofen 400 mg three times daily on the day of admission for four weeks.

**Figure 1:**
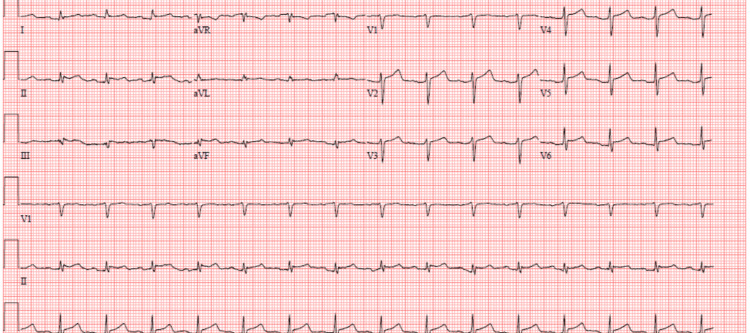
Baseline 12-lead ECG on admission showing ST changes and low voltage QRS complex

On day 2 of admission, an echocardiogram demonstrated a bright pericardium and a small pericardial effusion with a maximal depth of 0.9 cm. The ECG demonstrated marked ST changes and a low-voltage QRS complex, consistent with a pericarditis diagnosis (Figure 1).

On the advice of the cardiology team, at this time, clozapine was suspended, given its association with pericarditis. It is acknowledged that, in this case, the causative agent remains unclear and may well be viral or clozapine-related. Viral polymerase chain reaction (PCR) tests for influenza A, influenza B, and severe acute respiratory syndrome coronavirus 2 (SARS-CoV-2) were negative. On day 3, the medical review indicated no ongoing need for inpatient care, as chest pain had resolved and observations remained within normal parameters. The patient was transferred and admitted voluntarily to our psychiatric inpatient unit for consideration of ongoing management. On admission under psychiatry, it was noted that CRP and troponin had risen to 137 mg/L and 114 ng/L, respectively. This was discussed with the cardiology team, who felt that, given the clinical picture of stability, monitoring was all that was required. A repeat ECG on day 7 of admission demonstrated resolving ST elevation and T-wave inversion in keeping with the diagnosis of pericarditis. Multi-disciplinary discussions within the psychiatry team in the first days of admission concluded that the risks posed by the potential relapse of clozapine were more significant than the risk of clozapine-associated adverse effects. With input from the mental health pharmacist, the decision was taken to reintroduce clozapine once inflammatory markers and troponin had normalized and stabilized, provided the patient remained asymptomatic and clinically well. Blood tests, including CRP and troponin, were initially monitored every one to two days and were downtrending.

Clozapine was reintroduced on day 9 of admission, and at this time, troponin had fallen to 13 ng/l, and CRP was 36 mg/L. The patient had no complaints of chest pain or other symptoms and was clinically stable. Given the clozapine break of >48 hours and perceived increased risk in the context of pericarditis, a standard titration regimen over 15 days was followed as per local guidelines (shown in Table 1), and monitoring reverted to once weekly for six weeks. Troponin and CRP remained stable, though mildly elevated, throughout titration. Repeat ECG on day 7 of titration demonstrated no significant change from the previous.

**Table 1:** Dose titration and inflammatory marker trends during treatment CRP: C-reactive protein

Titration day	Dose	CRP (mg/L) (Normal <6)	Troponin (ng/L) (Normal <10)	White cell count (×10⁹/L) (Normal 4.0–11.0)
0	12.5 mg 6 pm	36	13	11.3
1	12.5 mg mane, 12.5 mg nocte	—	—	—
2	12.5 mg mane, 25 mg nocte	—	—	—
3	25 mg mane, 25 mg nocte	—	—	—
4	25 mg mane, 50 mg nocte	—	—	—
5	25 mg mane, 75 mg nocte	—	—	—
6	25 mg mane, 75 mg nocte	—	—	—
7	50 mg mane, 75 mg nocte	22	19	7.7
8	50 mg mane, 100 mg nocte	—	—	—
9	50 mg mane, 100 mg nocte	—	—	—
10	50 mg mane, 125 mg nocte	—	—	—
11	75 mg mane, 125 mg nocte	—	—	—
12	100 mg mane, 125 mg nocte	—	—	—
13	100 mg mane, 150 mg nocte	—	—	—
14	100 mg mane, 175 mg nocte	31	20	—
15	100 mg mane, 200 mg nocte	—	—	—
16	100 mg mane, 200 mg nocte	—	—	—
17	100 mg mane, 200 mg nocte	—	—	—
18	100 mg mane, 200 mg nocte	—	—	—
19	200 mg mane, 200 mg nocte	9	17	9.6

A repeat echocardiogram on day 15 of titration demonstrated an ongoing small pericardial effusion and nothing else of note. Clinically, the patient remained stable and asymptomatic throughout. There was no documented deterioration in the patient's mental state throughout this admission. The patient was successfully titrated up to a dose of 200 mg twice daily prior to discharge, expressing a desire to go back to the previous dosage of 700 mg daily. The decision not to quickly increase clozapine back to the previous dosing was based on the perceived increased risk given the context of pericarditis and the patient's stable mental state. He was discharged on day 31 of admission with community follow-up, including ongoing monitoring through the clozapine clinic. Although a small pericardial effusion persisted at discharge, the patient was asymptomatic. A repeat echocardiogram was reviewed with cardiology, who advised that no further cardiac follow-up was required. Post-discharge care included regular full blood count testing and outpatient psychiatric review. The patient has remained clinically well and asymptomatic six months post-discharge. A summary of key clinical events is presented in Table 2.

**Table 2:** Clinical timeline of events WCC: white cell count; CRP: C-reactive protein; MDT: multidisciplinary team

Day	Event
Day 0	Admission with chest pain; ECG showed widespread ST elevation; Labs: WCC ↑, CRP 16 mg/L, troponin 14 ng/L; Clozapine suspended; Started colchicine and ibuprofen.
Day 2	Echocardiogram: small pericardial effusion (0.9 cm), bright pericardium; ECG: persistent ST elevation, low-voltage QRS.
Day 3	Chest pain resolved; Transferred to inpatient psychiatry; CRP rose to 137 mg/L, troponin 114 ng/L; The patient remained stable.
Day 7	ECG: resolving ST elevation, new T-wave inversions; Clinically stable; MDT agreed on a potential clozapine re-challenge.
Day 9	Clozapine reinitiated at 12.5 mg; CRP: 36 mg/L, troponin: 13 ng/L; The patient was asymptomatic.
Day 9-23	Clozapine was titrated over 15 days per standard protocol; Serial CRP and troponin monitored; downward trend; ECG and echo remained stable.
Day 15 of titration (Day 23)	Repeat echocardiogram: small persistent effusion, no progression.
Day 19 of titration (Day 27)	CRP: 9 mg/L, troponin: 17 ng/L; WCC normalized.
Day 31	Discharged on clozapine 400 mg/day; Echo reviewed with the cardiologist: no further follow-up needed; Community monitoring via the clozapine clinic continued.
6 months post discharge	The patient remains asymptomatic and psychiatrically stable under community follow-up.

## Discussion

We present a case of successful clozapine re-challenge after pericarditis, highlighting the challenges and limited evidence surrounding this clinical scenario. Clozapine-associated pericarditis is a rare but potentially life-threatening side effect, and the decision to re-challenge patients with clozapine after such an event is complex. To date, only a few case reports have addressed the safety of clozapine re-challenge after pericarditis. Five cases have reported successful re-challenges, while others have not attempted re-challenges due to concerns about recurrent cardiac complications. Previous case reports have demonstrated varied outcomes. Kay et al. described a patient who developed pericarditis three weeks after initiating clozapine, yet continued treatment, achieving complete resolution of cardiac symptoms and maintaining psychiatric stability [5]. Similarly, Johal et al. (2019) reported a case where clozapine was continued without interruption despite the development of pericarditis after 15 years of treatment [6]. Boscutti et al. (2022), Crews et al. (2010), and Malhotra et al. (2002) also reported successful re-challenge with clozapine after pericarditis, with no recurrent cardiac complications [7-9]. However, not all re-challenge attempts have been successful. Daly et al. (1992) reported a case where the patient developed recurrent pleural effusion after re-challenge, leading to permanent discontinuation of clozapine [10].

Our case emphasizes the importance of a multidisciplinary approach, involving collaboration between psychiatry, cardiology, and pharmacy teams. Key factors contributing to the successful outcome included resolution of acute cardiac symptoms, normalization of inflammatory markers, gradual and cautious re-titration of clozapine, and close clinical and biochemical monitoring throughout. Additionally, patient factors, such as the absence of alternative effective antipsychotic options and the critical need for psychiatric stability, were central to the decision-making process.

Given the profound efficacy of clozapine in refractory psychosis and the serious consequences of psychiatric relapse, rechallenging clozapine after adverse cardiac events may be appropriate under selected conditions. However, clinicians must exercise extreme caution, ensure thorough risk-benefit discussions with the patient, and maintain vigilant monitoring post re-initiation.

## Conclusions

This case demonstrates that clozapine re-challenge following pericarditis can be safely undertaken in carefully selected patients with close multidisciplinary oversight and appropriate monitoring. Despite the rarity and potential severity of clozapine-associated pericarditis, our patient tolerated a gradual re-titration without recurrence of cardiac symptoms or biochemical deterioration. The decision to reintroduce clozapine was based on a thorough risk-benefit assessment, emphasizing the critical role of individualized care. This case supports the growing evidence that, in the absence of ongoing clinical instability, clozapine re-challenge may be a viable option for patients with treatment-resistant schizophrenia when alternative therapies are inadequate.
